# Why the Army Should Have a Veterinary Corps

**Published:** 1900-05

**Authors:** 


### EDITORIALS.

### WHY THE ARMY SHOULD HAVE A VETERINARY CORPS.

It is right that the army should be manned and officered by the men who are best adapted by nature and training for the work their country requires of them.

It is right that the army should have the most effective guns, ammunition, transportation facilities, equipment, and organization that can be devised and the country can afford.

All reasons for the very existence of the army are reasons for its most effective equipment and organization. It is the duty of the government to the army and the country to provide means for the utilization of the best knowledge and practice extant in so far as relates to the economy and preparedness of the army in time of peace and its comfort and efficiency in time of war.

*Mobility* is frequently the factor determining success or failure. The requisite degree of mobility can be had only with adequate transportation facilities. Transportation in the field and at the actual seat of war depends largely, in some instances solely, on animals—horses, mules, and oxen. The cavalry and light artillery depend upon horses almost as much as upon men.

It is essential not only to economy, but to efficiency as well, that the horses should be well selected; that they should be sound and suitable for the work they have to do; that their needs should be thoroughly studied, understood, and met; and that they should, as far as possible, by the application of the best methods known, be maintained in strong, serviceable, and healthy condition.

The army should have the advantage of the services of men competent to regard those questions from the stand-point of the animal engineer. Such men must be thoroughly drilled and proficient in the application of the sciences of animal anatomy, physiology, chemistry, hygiene, medicine, and surgery. They should be skilled in the principles and practices of horsemanship; they should have an intimate knowledge of the chemistry, the nutritive values and the defects of all kinds of forage; of the proper care of animals under all conditions; of the prevention and remedy of defects and disease. This knowledge is extant and taught in such universities as Harvard, Cornell, the University of Pennsylvania, and the State Universities of Iowa, Ohio, and California to students who graduate as veterinarians. Such men are animal doctors and animal engineers.

With the single exception of the United States, the army of every civilized country in the world has found it economical and advantageous to accept the benefits that come from the development and application of veterinary knowledge.

In this country the army has never had the benefits of this knowledge, because the positions for veterinarians have been such that self-respecting men of ability could not accept them. The result has been that, with very few exceptions, veterinary science has been misrepresented by a lot of incompetent men who have failed in civil practice and have gone into the army because the regimental commanders who have hired them have not recognized the scope, possibilities, or worth of veterinary services, and have thought that anyone willing to do such work as they have required is good enough to be an "army horse-doctor." These civilian employés attached to the army have been without instruments, supplies, or suitable regulations, to say nothing of dignity or authority. The very few competent veterinarians who have drifted into the army have resigned after discovering the impossible conditions governing them, or are waiting for the enactment of a law that will place their work on a proper basis.

If the army and the country are to have the full benefits of the best knowledge extant in relation to the care and utilization of animals, and if the horses and mules used in the cavalry and artillery arms of the service and in the transportation department are to be maintained with the greatest attainable economy and in the highest efficiency, it is clearly necessary that the best veterinarians shall be employed in positions where they can make their work count. To continue to employ veterinarians without rank or official authority is of little value. To be sure, better men will be selected under the recent regulation requiring applicants to pass an examination; but however good the individuals, their work will not, under the present faulty system, give the results the public expects and has a right to demand.

The veterinary profession has been so sadly misrepresented in the army for such a long time, and veterinary science has, in consequence, been so frowned upon and repressed, that it will be difficult for it to assume its proper position.

That certain high officials in the War Department have absolutely no conception of what veterinary science is and what it is for is shown clearly by an incident of the war with Spain. When it was proposed to invade Cuba, and many thousand horses and mules were gathered at a camp in Florida, several contagious diseases, including glanders, prevailed among these animals. If the original plans had been carried out it would have been of the highest importance to have had the cavalry and artillery horses and the train mules sound and efficient. A few veterinarians of unknown antecedents or experience were picked up and asked to treat the sick animals. They were not supplied with the drugs or instruments needed, and could not get them, because there was no one in the War Department who was capable of realizing their needs. They had no authority, and private soldiers caring for animals could obey their advice or not, as they preferred. Of course, things did not go well; the most simple precautions were disregarded and glanders spread at an alarming rate. In this grave crisis an itinerant vendor of patent medicines and horse-boots was selected by an officer in the War Department and placed in charge of the sick animals as "chief veterinarian." This individual was ignorant of veterinary science, and was helpless. The two most efficient veterinarians previously employed there, either one of whom could have eradicated the threatening scourges, could not work under the direction of an overbearing incompetent, so both resigned. The Bureau of Animal Industry of the Department of Agriculture was finally called upon for assistance. This shows that when certain individuals say that a veterinary corps is unnecessary they do not know what a veterinary corps is or what it can do. They have no conception of veterinary science. The fact that the best armies in the world have such a corps has no significance to them, because they cannot see beyond the kind of so-called veterinary service the army has always had.

The corps organization is needed because without it the veterinary work will never be able to assume its true position of usefulness. Without a corps, army veterinarians will have no standing in the War Department; and their recommendations, filtering in through unsympathetic channels, will be emasculated and deprived of all value. Without a corps the work will be unorganized and unsystematized, and will fail to give better results than can be obtained by each individual regimental veterinarian. Whereas, with a corps the whole work can be raised to the standard of the chief veterinarian—who ought to be one of the best veterinarians in the country. Without a corps the veterinary work of the army will, by admission of the War Department, be placed in charge of a fourth-class clerk, thus demonstrating that an influential element in the War Department means that the army shall not have the advantage of adequate veterinary service. Of course, it is not meant that there is any direct antagonism to increased efficiency along veterinary lines; but the ignorance on this subject is so profound that it appears to be absolutely necessary that an organization having standing and power shall be created to bring into being the veterinary reforms that the army needs.

This country has always stood for progress. It has always been foremost to accept and utilize available knowledge. Its attitude toward veterinary science is no exception to this rule. The Bureau of Animal Industry is under the direction of a chief who is a veterinarian. This Bureau has made discoveries in animal pathology that have been of inestimable value to the country. In its executive work it has succeeded in effecting sanitary results that have challenged the admiration of the world. It has eradicated contagious pleuro-pneumonia of cattle in a shorter time and at less expense than have marked the completion of this work in any other country in the world. It is upon the veterinary work of this Bureau that the export trade of the United States in animals and animal products depends. This Bureau expends and gives a good return for almost $1,000,000 per year.

Almost every State in the Union has a State veterinarian, who holds a position of responsibility and trust. Veterinary schools have been erected and are maintained by several States and universities because the value of the veterinary science is manifest and recognized. It is only in the army, and, of all armies, only in the army of the United States, that veterinary science is not appreciated as a useful science and veterinarians are not recognized as useful men, deserving of positions in which they can show the value of their knowledge and work. The present feeling toward this proposed reform may be compared to the opposition that had to be overcome by the engineers of the navy when it was proposed to recognize them as officers. But the opposition was mastered, the reform came, and has been amply justified. So it is in regard to veterinary science—to animal engineering. The reform will come. All history shows this, and the sooner this service is placed on an appropriate and adequate basis the sooner the nation will reap the reward in increased economy and efficiency of her army.

